# Role Clarification Processes for Better Integration of Nurse Practitioners into Primary Healthcare Teams: A Multiple-Case Study

**DOI:** 10.1155/2014/170514

**Published:** 2014-12-01

**Authors:** Isabelle Brault, Kelley Kilpatrick, Danielle D'Amour, Damien Contandriopoulos, Véronique Chouinard, Carl-Ardy Dubois, Mélanie Perroux, Marie-Dominique Beaulieu

**Affiliations:** ^1^Faculty of Nursing, University of Montreal, P.O. Box 6128, Centre-Ville Station, Montreal, QC, Canada H3C 3J7; ^2^University of Montreal Public Health Research Institute, P.O. Box 6128, Centre-Ville Station, Montreal, QC, Canada H3C 3J7; ^3^Department of Family Medicine and Emergency Medicine, University of Montreal, CRCHUM, Saint-Antoine Tower, 850 St. Denis Street, Room S03-284, Montreal, QC, Canada H2X 0A9

## Abstract

Role clarity is a crucial issue for effective interprofessional collaboration. Poorly defined roles can become a source of conflict in clinical teams and reduce the effectiveness of care and services delivered to the population. Our objective in this paper is to outline processes for clarifying professional roles when a new role is introduced into clinical teams, that of the primary healthcare nurse practitioner (PHCNP). To support our empirical analysis we used the Canadian National Interprofessional Competency Framework, which defines the essential components for role clarification among professionals. A qualitative multiple-case study was conducted on six cases in which the PHCNP role was introduced into primary care teams. Data collection included 34 semistructured interviews with key informants involved in the implementation of the PHCNP role. Our results revealed that the best performing primary care teams were those that used a variety of organizational and individual strategies to carry out role clarification processes. From this study, we conclude that role clarification is both an organizational process to be developed and a competency that each member of the primary care team must mobilize to ensure effective interprofessional collaboration.

## 1. Introduction

High-performing primary care teams are one of the chief characteristics of healthcare systems that are responsive to population needs [1, 2]. However, the optimal development of such teams is a challenge for primary care systems. Optimizing each professional's scope of practice is one suggested approach to reinforce team functioning and respond to patients' needs [3]. This is difficult, however, because changes in professional roles can lead to power struggles [4]. Whenever the nurse practitioner role has been introduced into clinical teams, one of the greatest difficulties encountered has involved the clarification of professional roles [5]. Lack of clarity about the nurse practitioner's role has created confusion and led to resistance to its integration [5, 6]. Clarifying professional roles among members of a primary care team can be an effective approach to mitigate power struggles, facilitate the integration of new roles in teams, and foster interprofessional collaboration.

In primary care practice, there is a great deal of role overlap, specifically with regard to the roles of physicians, primary healthcare nurse practitioners (PHCNPs), registered nurses, and other professionals on the clinical teams. Clarifying professional roles can serve several purposes: defining each person's responsibilities, ensuring appropriate implementation of each professional's role, optimizing professional scopes of practice, and thereby ensuring efficient patient management.

PHCNPs were introduced in Quebec in 2008 to provide health and wellness promotion and to treat patients requiring follow-up for an acute common illness, chronic disease management, and pregnancy follow-up to 32 weeks of gestation. In Quebec, PHCNPs are Master's prepared registered nurses. PHCNPs can order and interpret diagnostic tests, prescribe medication and medical treatments, and perform specific procedures within their legislated scope of practice [7]. The focus of the PHCNP role is health promotion, disease prevention, preventive care, diagnosis of acute minor illness and injuries, and monitoring and management of stable chronic conditions [8]. PHCNPs in Quebec have some restrictions on their scope of practice, including their ability to establish a primary diagnosis [8].

Several researchers have explored collaboration between physicians and nurses in primary care teams [9], but, to our knowledge, no study has specifically examined the processes by which professionals clarify their respective roles in these teams. In this paper, our objective is to examine the processes of role clarification among professionals in primary care teams when a new role is introduced into the team, that is, the PHCNP. Specifically, we want to understand the organizational and individual components that influence the process of professional role clarification. In the following section we present the key elements of the Canadian National Interprofessional Competency Framework.

## 2. The Canadian National Interprofessional Competency Framework 

The Canadian National Interprofessional Competency Framework was developed by the Canadian Interprofessional Health Collaborative (CIHC) in 2010. The CIHC is made up of academics, researchers, health professionals, students, and health organizations concerned with training for interprofessional collaboration and the associated competencies. This framework defines the competencies required for better collaboration; it positions role clarification as one of the fundamental competencies for optimizing interprofessional collaboration [10]. This framework is relevant for two reasons. First, it presents a shared vision of the competencies associated with interprofessional collaboration. Second, it allows us to link specific activities with the implementation of the role clarification competency, to gain a better understanding of the processes involved. The CIHC framework and the role clarification competency are briefly described below.

According to the CIHC [10], interprofessional collaboration is encompassed by six competency domains: (1) interprofessional communication, which refers to effective, responsible, and open communication within the interprofessional team; (2) patient/client/family/community-centred care, which examines the involvement of patients and their families in care planning; (3) team functioning, which reflects the importance of knowing the mechanisms and principles underpinning effective team functioning; (4) collaborative leadership, whose aim is to facilitate shared decision-making and develop a collaborative work climate by applying the principles of consultation to decision-making; (5) interprofessional conflict resolution, which seeks to engage all team members in identifying constructive solutions to any conflicts arising within the interprofessional team; and (6) role clarification, which is presented in greater detail in the following paragraphs. These competencies are mobilized differently depending on the practice context and the complexity of clinical cases, as well as on the quality improvement processes in place in the clinical settings.

Besides the competencies that underlie interprofessional collaboration, the CIHC framework [10] puts forward three other components to be considered when interprofessional teams work together: the complexity of clinical situations, the practice context, and quality improvement. These components influence the application of the competencies, as they may determine the intensity of interprofessional collaboration in clinical teams. The* complexity of clinical situations* refers to those situations in which professionals collaborate. They cover a broad spectrum ranging from simple to complex. In most cases, complex situations require the involvement of many professionals. The* practice context* influences interprofessional collaboration. Indeed, depending on the setting, care teams function differently and adapt differently to the needs of patients and families.* Quality improvement* is addressed more effectively using interprofessional collaboration. All actors in the healthcare system must work together to transform practices and to improve the quality of care and services. These three components (i.e., complexity of clinical situations, practice context, and quality improvement) refer essentially to elements of the organizational context in which care is provided.

### 2.1. Role Clarification: An Interprofessional Collaboration Competency

The CIHC framework describes the role clarification competency as follows:

*Learners/practitioners understand their own role and the roles of those in other professions, and use this knowledge appropriately to establish and achieve patient/client/family and community goals. [10] (page 12)*

This competency is closely linked with the other five competency domains identified in the CIHC framework and is aimed at supporting processes to optimize each professional's field of practice. It is based on a detailed understanding of one's own role and those of other professionals. This competency, which is fundamental to interprofessional collaboration, is defined by seven descriptors that identify the relevant knowledge, attitudes, and values, which are dynamic and constantly evolving [10]. These role clarification competency descriptors include the following:

1. describing own role and that of others;
2. recognizing and respecting the diversity of other healthcare and social care roles, responsibilities, and competencies;
3. performing own roles in a culturally respectful way;
4. communicating roles, knowledge, skills, and attitudes using appropriate language;
5. accessing others' skills and knowledge appropriately through consultation;
6. considering the roles of others in determining own professional and interprofessional roles;
7. integrating competencies/roles seamlessly into models of service delivery.

In order to better understand the role clarification process, it is important to examine the components of the context that influence role clarification (complexity of clinical situations, practice context, and quality improvement) as well as the seven descriptors of role clarification presented above.

## 3. Methods

### 3.1. Research Design

This descriptive multiple-case study [11] was approved by the Research Ethics Board of the University of Montreal. Case study design was recommended because it allows for in-depth examination of events while taking into account the larger context in which they occur [12]. The CIHC Framework was used as a reference model to direct data analysis [13]. Multiple-case study design is more robust because using a framework allows the researcher to compare and generalize findings to other cases (i.e., analytic generalization) [11].

### 3.2. Case Selection and Description

Each case was operationally defined [14] as the primary healthcare setting where the PHCNPs practiced. The cases were bounded by the limits of service as determined by the PHCNP's practice model and reporting structure. We used purposeful sampling [15] to identify cases that offered a broad picture of PHCNPs' roles in the province of Quebec, Canada, where PHCNP role implementation is recent. The cases were selected based on their similarities and differences in terms of geographic location and time elapsed since implementation. We identified six cases in four geographic regions of the province. Half the cases were situated in predominantly rural catchment areas. PHCNPs served patients in all age groups. Also, the cases present a diversity of characteristics in terms of patient management models, size of interprofessional teams, number of professional staff, and number of patients seen by the PHCNP. These intercase variations provide a wealth of data to better understand the phenomena under study [16] which are the deployment of PHCNPs in primary care settings and the associated role clarification processes. The patient populations under PHCNP care included patients without a regular physician, new immigrants, and refugees, among others; conditions managed included chronic illnesses such as diabetes or hypertension, mental health conditions, pregnancy, and routine acute health problems such as otitis. The cases studied are described in Table 1.

### 3.3. Data Collection and Participants

Data were collected from May 2011 to October 2011. We conducted 34 individual interviews: 11 nurse managers or nursing directors, 15 intraprofessional (i.e., within nursing) team members, seven physician partners, and one interprofessional (outside nursing) team member (Table 2). In each setting, we interviewed the key actors directly involved in the PHCNP integration processes, who were mainly PHCNPs, physician partners, and nurse managers. In only one case was a professional other than a nurse involved in the implementation of the new PHCNP role in the primary care team.

We used semistructured interview guides [17], which we adapted according to the professional's role (e.g., nurse manager, physician, PHCNP, and registered nurse). Initial questions and themes were identified from the literature and included questions about the preparation of the work setting, implementation of the PHCNP role, their vision for the PHCNP role and that of the team, the activities comprised in the PHCNP role, how activities were shared between PHCNPs and other team members, and collaboration within the team [8]. The interviewer's questions were flexible to allow exploration of emerging themes as the interviews unfolded [18]. Interviews were generally audio-recorded with participants' permission and transcribed. For those not audio-recorded, summary notes were prepared. Interviews lasted between 25 and 75 minutes.

### 3.4. Data Analysis

The overall aim of the analysis was to understand how roles were clarified among team members. The interviews were analyzed within the context of the case, and then the findings from each case were compared. All the interviews were read several times by different members of the research team “to obtain a sense of the whole” (page 108) [19]. Each site was given a unique identifying code number to facilitate retrieval [20]. An overall summary of each case was developed by the research team member responsible for the case to identify key themes.

#### 3.4.1. Phase One: Within-Case Analysis

The qualitative data was analyzed using qualitative content analysis [19], defined by Hsieh and Shannon as the “subjective interpretation of the content of text data through the systematic classification process of coding and identifying themes or patterns” (page 1278) [21]. We used the approach described by Miles and Huberman [20] to organize and synthesize the collected data. Matrices were used to display the data and identify patterns [20].

To gain a better understanding of the role clarification process [22, 23], our analysis was supported by the descriptors of the CIHC role clarification competency. We sought to populate the role competencies framework [20, 24]. No additional codes were created. Langley argued that, to understand processes, it is essential to “document as completely as possible the sequence of events” (page 692) [25]. We identified instances where participants described how activities or perceptions had changed over time. Subsequent to this step, we explored similarities and differences in processes within and across the cases [9].

#### 3.4.2. Phase Two: Cross-Case Analysis

In phase two, we proceeded to the overall analysis across the six cases [11]. We used inductive and deductive approaches [26] to understand how roles were clarified. Similarities and differences in role clarification were identified across cases. To identify patterns across the cases, we developed a matrix of the themes and concepts identified in the within-case analysis [20]. We used pattern-matching to understand how the cases fit within the CIHC framework [20].

### 3.5. Rigour

We used several strategies to ensure the quality of the case studies [11]. We collected data from different sources (PHCNPs, physician partners, and nurse managers) and from rural and urban regions of the province. Patterns in the data were identified by using the concepts of the CIHC framework. We compared the findings across the cases to understand how the process of role clarification occurred [11]. Case study methodology uses analytic generalization to generalize findings to a broader theory [11]. The explicit description of the methodological approach is intended to facilitate reproduction of the study. However, any application of the results must take into consideration the particular features of other contexts [16]. In this study, the identification and selection of multiple cases allowed us to identify similarities and differences across the cases for anticipated reasons, thereby strengthening the conclusions [27]. Through the interviews, we were able to gather information on the different concepts associated with the study, and we conducted interviews until we reached the point of data saturation, when information provided in new interviews was redundant [16]. The results were validated with the participants in the majority of the case settings.

## 4. Results

The results are presented here in reference to the Canadian National Interprofessional Competency Framework. First, we present the empirical results with respect to the three organizational components that influence interprofessional collaboration: the practice context, the complexity of clinical situations, and quality improvement. This initial step is helpful in understanding how these elements influenced role clarification. We then look specifically at the role clarification competency and present our results in relation to the seven descriptors of this competency in order to better understand how this competency was mobilized in the different settings studied.

### 4.1. The Practice Context

Our empirical data showed that, with respect to practice context, clarification of the PHCNP's role was based on two elements: an analysis of unmet patients' needs and the legislative framework governing PHCNP practice in Quebec. Accessibility to healthcare and services was the key element that emerged from the analysis of patients' needs. In the high-performing settings, this needs analysis preceded the arrival of the PHCNP and helped clarify the PHCNP's role in the primary care team:

*Here in our organization, we met with the director of nursing to determine which would be the most appropriate sectors to receive a PHCNP, and what roles she might take on, while also taking into account the regulations of the regional health agency. (Clinical nurse specialist)*

The analysis of patients' needs and the clarification of the PHCNP's role in the team were directly based on knowledge of the legislative framework governing PHCNP practice, in which the scope of the PHCNP role, prescribing activities, and eligible practice settings are defined. Settings that were less knowledgeable about the PHCNP's scope of practice encountered problems in assigning patients to the PHCNP. For example, if the assigned patients had complex needs that could not be entirely addressed within the PHCNP's scope of practice, this inhibited the autonomy of the PHCNP's role. The high-performing settings used the legislative framework to negotiate the PHCNP's role with the different members of the primary care team, such as, among other things, to confirm with pharmacists and radiologists the PHCNP's right to prescribe in that setting.

Thus, the practice context refers to the local context of care and services for each of the settings studied. The legislative context also influenced role clarification, as it helped define the different practitioners' scope of practice (e.g., pharmacist and radiologist).

### 4.2. The Complexity of Clinical Situations

Clinical complexity refers to the broad spectrum of situations encountered by PHCNPs and primary care teams, ranging from simple to complex. The complexity of clinical situations creates opportunities for interaction among the different members of the primary care team. Most of the settings had instituted formal occasions for interaction through team meetings. Such meetings fostered role clarification among team members and primarily between the PHCNP and the physician partner. However, in some settings, physicians met only among themselves and discussed the PHCNP's practice and how the PHCNP could be more effective and better used:

*I think each FMG [family medicine group] will develop, to some extent, its own modus operandi with the nurse practitioner regarding what they want her to do for chronic illness management. *(Physician partner)

In another setting, meetings were held to clarify roles in relation to the preferences and interests of the PHCNP:

*I sat down with the PHCNP to talk with her about her preferences, her wishes, what she hoped to do in coming to work at our clinic, because the needs are so great that we have to make choices. (Physician partner)*

The complexity of clinical situations is an issue to be considered when new PHCNPs are introduced and when the PHCNP's caseload is created. Our analysis showed that when clinical situations were at the high end of the spectrum in terms of complexity, PHCNPs needed to consult physician partners more often. In settings that implemented a system in which nursing assistants collected preliminary patient data to assign patients by level of complexity (two cases in six), the number of consultations between PHCNPs and physician partners was greatly reduced. This initial screening made it possible for PHCNPs to target patients whose full range of healthcare consultation needs they could manage:

*… the added value of a PHCNP is that she rarely needs to consult the physician partner; that's when it becomes worthwhile. (PHCNP)*

### 4.3. Quality Improvement in Care and Services

Integrating a new member into the primary care team can create opportunities to improve the quality of care and services. The introduction of a PHCNP led to a renewed vision of nursing in primary care in which the roles of the different types of nurses could be optimized:

*… it's having a vision for nursing practice, saying, what roles should we be giving to each of these different job categories in nursing? So, not to think only about the PHCNP, and where we should put her, but to ask what role she should play in relation to the role of the registered nurse, the role of the staff nurse. But for me, that's always what I'm thinking about, because I also have a mandate, an organizational responsibility, which is to optimize care, or to set care priorities. (Nursing coordinator)*

Having clearly defined roles in primary care teams also makes it possible for new professional projects to emerge. Some settings had implemented special projects led by the PHCNPs. These projects helped to promote the PHCNP's role in the team and to underscore the specificity of that role and how it differed from the physician's role. Our data showed that this type of project had positive impacts on the clinic's overall performance:

*First, we started by meeting them [PHCNPs]. It's a little simplistic to say. Then to … learn from them what were their abilities, their limitations, and … their wishes to … Not their wishes, but what they wanted to do as work, and what they were able to do as work. (Physician partner)*

### 4.4. Role Clarification Competency Descriptors

As mentioned earlier, the Canadian National Interprofessional Competency Framework presents seven descriptors for the role clarification competency. The analysis of our data provided a better understanding of how the descriptors of this competency were manifested in the primary care teams. In this section we present our empirical results in relation to these descriptors.

(*1) Describing Own Role and That of Others*. Role clarification in a primary care team requires a detailed understanding of one's own professional role and those of others. In most teams, the arrival of a PHCNP meant integrating a new professional role into the team. PHCNPs have a key responsibility for clarifying their role within primary care teams. The majority of our respondents said PHCNPs were the best persons to explain their own role to other team members. However, PHCNPs could also find themselves in a vulnerable position if their role in the team is a new one and might need the support of resource persons in the setting to support role clarification. Such support from a resource person is crucial to communicate the role optimally and to ensure it is well understood by all the team members. Several respondents estimated that it took about six months for PHCNPs to settle into their role and up to a year for all dimensions of that role to be fully integrated. According to one PHCNP and a physician,

*… you have to build up trust, a new role … And no one knows the role better than we do. The guidelines, physicians, they have an idea of the role, but … I had to tell them, “okay, I've come to see you [physician] because I think the patient needs this drug, but I cannot prescribe it.” (PHCNP)*

* Ah! OK. You know he needs it but you cannot prescribe it? OK. (Physician partner)*

Nevertheless, once the PHCNPs had settled fully into their role, some respondents were very satisfied with their practice:

*… I was pleasantly surprised by their—how could I say it?—their capacity for work and for managing interprofessional care plans. So I quickly accepted this principle and this form of team. (Physician partner)*

(*2) Recognizing and Respecting the Diversity of Other Healthcare and Social Care Roles, Responsibilities, and Competencies*. Role diversity refers to all the dimensions associated with a professional role and its responsibilities and competencies. The diversity of the PHCNP's role is not very widely recognized in primary care teams. Sometimes roles are differentiated uniquely on the time allocated for patient consultations, which varies from one professional to another. As this respondent pointed out,

*The PHCNP's consultation will be more comprehensive. Physicians know that, in her role, the PHCNP can take more time for consultations with patients. Also, the nursing dimension of the role makes the difference in terms of teaching. (Clinical nurse specialist)*

Another respondent sees the diversity of the role in a positive light and respects the difference:

*I don't have any genograms in my patients' charts. The PHCNP does, and for her it's a value, which I respect. But at the same time, for us doctors, we need to respect the fact that the PHCNP won't be doing things exactly the same way we do them. (Physician partner)*

(*3) Performing Own Roles in a Culturally Respectful Way*. This competency indicator can take various forms: first, as respect for the cultures of the different professionals in the teams and then as respect for the culture and values of patients and families followed by the primary care teams. Several PHCNPs in our cases worked with recent immigrants from other countries, refugees, and vulnerable and impoverished groups. The PHCNPs described how they incorporated culturally adapted strategies in their diabetes teaching activities.

As an illustration of this competence, in the less well performing settings, professionals in the same discipline, for example, the PHCNPs and nurses, tended to stick together for mutual support. These alliances occurred more frequently in settings where there was less collaboration with physicians, and they were a source of comfort and support for the PHCNPs. 

(*4) Communicating Roles, Knowledge, Skills, and Attitudes Using Appropriate Language*. Our data showed that members of primary care teams used several different means to communicate their roles, knowledge, abilities, and attitudes in appropriate language. In some of the settings studied, the PHCNP's role was explained to the primary care team in a presentation made by the nursing director and the PHCNP together, in which they were able to respond to various questions from the team members.

In most of the settings, care team members primarily used informal communication to discuss professional roles. Setting size also influenced the type of communication used. Settings with fewer professionals tended to opt for informal communication, whereas settings with larger teams tended to schedule formal sessions for communication. In our study, the better performing settings were those where there was ongoing communication, formal and informal, among the members, in which they were able to discuss grey areas and each person's capacities.

One setting formalized the process of role clarification by developing a matrix of all team members' roles. This matrix was a tool for discussing areas of overlap among the professionals and enabled team members to clarify each person's expertise. As one PHCNP reported,

*So, at that moment, the roles were well defined; it helped. I think that, for sure, it doesn't solve all the problems. Having it on paper, you can refer to it, it's useful, but afterward, it's mostly in talking with people. (PHCNP)*

The settings that used only documents prepared by the professional nursing and medical associations to clarify roles said these were insufficient and that interactions with other members of the team were essential. 

(*5) Accessing Others' Skills and Knowledge Appropriately through Consultation.* We identified two types of patient management models in the cases: the joint management model and the consultative management model. In the joint management model, a group of patients is followed jointly by both the PHCNP and a physician partner. In the consultative management model, they each manage their own group of patients, and the PHCNP consults the physician as needed for the patients she is following. The consultative model was used in the majority of settings studied. This patient management model fosters consultations between the physician partner and the PHCNP and requires that time be set aside for such exchanges, as pointed out by this PHCNP:

*We planned specific times when we could meet: early in the morning, at lunch time, and at the end of the day. These were our times when we talked about cases. (PHCNP)*

This type of consultation did occur not only between physicians and PHCNPs, but also between nurses and other team members. However, our data revealed that the informal nature of the consultations sometimes made the PHCNPs uneasy, as they worried about bothering their colleagues, specifically physicians, and disrupting their work. 

(*6) Considering the Roles of Others in Determining Own Professional and Interprofessional Roles*. In primary care, PHCNPs work closely with physician partners to establish their place and their complementary role in the team. This involves identifying what is unique about the PHCNP's role and what elements of the role are shared with other professionals, that is, certain tasks that can also be performed by other professionals or competencies that can be shared by more than one member of the team. According to one physician, the sharing of clienteles raises issues about the physicians' medical role:

*We're only going to have the complicated cases, the cases no one wants. Our more stable, easier cases will be given to others. So there's a sort of reassessment happening among the professionals, particularly with respect to the PHCNPs' role. (Physician partner)*

For the nurses, the arrival of the PHCNP provoked some apprehension. Some of them had developed expertise in managing patients with diabetes and were worried they would lose that role when the PHCNP arrived. Other nurses saw the PHCNP's arrival as an opportunity to ensure better management of their clienteles and as providing an additional resource to which they could refer patients as needed. According to one PHCNP, recognizing and maintaining the expertise of registered nurses was a key element in defining the PHCNP's own role:

*The registered nurses have expertise in adjusting insulin and in the very hands-on management of hypertension. Knowing this from the start made it possible for them to keep their expertise, and for us to take our own place. That was a really, really positive thing. (PHCNP)*

The PHCNP's arrival was a catalyst for redefining the roles of every member of the primary care team. According to this nursing director,

*They asked me to review roles and to optimize the roles of registered nurses. So we reviewed the roles of the licensed practical nurses, who were doing more than they were supposed to be doing. We reviewed the roles of the registered nurses, who weren't doing enough. (Director of Nursing)*

In several settings, the PHCNP's role was developed by building on the complementarity of roles already existing in the team. 

(*7) Integrating Competencies/Roles Seamlessly into Models of Service Delivery*. In several settings, integrating PHCNP competencies into new models of care delivery resulted in the creation of specific clinics for PHCNPs, such as teams for the prevention and treatment of sexually transmitted and blood-borne infections (STBBI). This type of clinic provided an opportunity for PHCNPs to promote their role within the establishment and not just in the primary care team and to showcase the distinctive characteristics of the PHCNP's role in comparison with the role of physicians. These special projects extended outside the boundaries of the primary care clinic; they were projects that involved the entire organization and allowed service delivery to be modulated based on the PHCNP's competencies.

In another setting, the PHCNP was a core resource for training nursing staff, in order to achieve optimal integration of collective prescriptions among nurses (e.g., adjusting antihypertensive medication):

*The new director's vision consisted of fostering the autonomy of each professional role. To achieve this, implementing and developing collective prescriptions was essential. (PHCNP)*

The arrival of the PHCNP was thus an opportunity to optimize services to the population. The less well performing settings were those that did not incorporate any special projects into the PHCNP's work.

## 5. Discussion

From the analysis of our empirical data, two major conclusions emerged regarding role clarification in primary care teams: role clarification is both an organizational process and a professional competency. Overall, half of the settings studied (3/6) were defined as “high-performing,” in that they had successfully integrated the PHCNP into the primary care team, there was clarity and consensus among team members about their roles, and interprofessional collaboration was well established within these teams.

### 5.1. Role Clarification: An Organizational Process

In the settings studied, the best performing teams were those that introduced organizational processes to support role clarification. The organizational processes could take several forms: developing a matrix to clarify professional roles, allocating formal time for discussing roles, or implementing special projects for PHCNPs, such as STBBI clinics. According to D'Amour and colleagues [28], these elements correspond to the dimension of formalization of interprofessional collaboration through the use of tools and information exchange. Our data suggest that, for optimal results, role clarification needs to be planned. At the level of nursing administration, in settings where a “champion” was designated to oversee optimal implementation of the PHCNP role and make it known to others before the PHCNP's arrival, the integration was facilitated and colleagues had a better understanding of the role. Several researchers have emphasized the key role of nursing leaders in implementing new nursing roles. Their understanding of these roles affects the implementation of all aspects of the role [6, 8, 29, 30].

### 5.2. Role Clarification: A Professional Competency

The second conclusion emerging from this study is that role clarification is a competency professionals need to mobilize to ensure their own role is well understood by all team members. As such, professionals have the responsibility for fully understanding their own role and the various dimensions associated with their practice, so they can explain it to the team, make the case for it, and negotiate accordingly. The professionals need to identify all the ways in which the mobilization of this role clarification competency is manifested. The best performing settings were those in which individuals were able to talk about their own roles and understand those of other professionals. This paper provides a first empirical application of the Canadian National Interprofessional Competency Framework and contributes to a better understanding of the development of the interprofessional collaboration competency and more specifically of the role clarification competency. To facilitate the appropriation of the different descriptors associated with role clarification and reduce overlap, these descriptors could be reduced to four. Several interview comments could have been encoded in more than one competency descriptor (e.g., Competency 1: describing own role and that of others and Competency 6: considering the roles of others in determining own professional and interprofessional role). Research is needed to further clarify the distinction between these competencies. Our research has shown that applying the Canadian National Interprofessional Competency Framework empirically is sometimes difficult. Some data fell within more than one descriptor at a time. Nevertheless, using the CIHC framework was helpful in developing a better understanding of the role clarification competency and thereby fostering its full deployment.

## 6. Practical Implications

This study has provided a deeper understanding of the processes of role clarification when the PHCNP role is integrated into primary care teams. A limitation in case studies concerns the ability to generalize the results to larger contexts, since the cases studied are situated in particular contexts. It is nevertheless possible to draw some practical implications, as presented here.

Effective role clarification processes are those that include both an organizational dimension, in which processes are set up to facilitate role clarification, and an individual dimension, in which professionals are able to communicate clearly all aspects of their roles. All the settings agreed that role clarification processes must take into consideration patients' needs. Role clarity is a key determinant of interprofessional collaboration. Our data showed that no team had planned for a systematic role clarification process in a given time and space. Nevertheless, according to Carmel and Baker-McClearn [31], professional roles are dynamic and become transformed by the practice context and by interactions with other professionals. These authors consider that roles are constructed and negotiated by means of the everyday processes experienced by members of the team. The roles are defined not only by these interactions, but also by legislation and by professional regulatory agencies [31]. For these reasons, roles are not static; they evolve with patients' needs, providers' experience with the role, technological developments, training received, and legislation, and as such, they need to be redefined over time. Therefore, it would be important, as the years go by, to set aside time and space to discuss how roles are changing in primary care teams.

In conclusion, our aim in this paper has been to gain a better understanding of role clarification processes in primary care teams in Quebec. Role clarification is a key determinant of interprofessional collaboration, with both an organizational and a professional component. As such, it is the responsibility of both the organization and the primary care team members. Interprofessional collaboration is a complex competency for clinical teams to acquire, and role clarification is one of the essential competencies they need to master.

## Figures and Tables

**Table 1:** Description of the cases.

Theme	Cases					
	Case 1	Case 2	Case 3	Case 4	Case 5	Case 6
Location	Urban	Rural	Rural	Rural	Urban	Urban

Patient management model: joint, consultative, mixed^*^	Mostly consultative	Mixed	Consultative	Mixed or consultative	Consultative	Exclusively consultative

Type of clientele (socioeconomically, geographically). Large territory refers to a geographical area covering more than 3 cities and hundreds of miles	Varied clientele, socioeconomically poor Small, dense territory	Clientele with chronic illnesses Large territory	Economically disadvantaged clientele High birth rate Large territory	Clientele with chronic illnesses Pediatric clientele Large territory	Clientele with chronic illnesses, socioeconomically poor, immigrants Small, dense territory	Home care clientele (with the exception of palliative care) Small, dense territory

Type and number of professionals working closely with the PHCNP	2 PHCNPs, 8 MDs, 2 RNs, 1 nursing assistant, 1 social worker, 1 psychologist, 1 nutritionist, 1 kinesiologist	1 PHCNPs, 5 MDs, 2 RNs, 1 nursing assistant, 1 nutritionist	2 PHCNPs, 2 MDs, 4 RNs, 2 nursing assistants, 3 social workers, 1 occupational therapist	2 PHCNPs, 2 MDs, 1 RN, 1 nursing assistant	1 PHCNP, 1 MD, 1 RN, 1 nursing assistant, 1 pharmacist	1 PHCNP, 3 MDs, 2 RNs, 2 social workers, 2 occupational therapists, 2 physiotherapy technicians

Patients seen by the PHCNP on a daily basis in the walk-in clinic	Around 8–10 patients/day	5-6 patients/day	9 patients/day	Around 9 patients/day	Around 9 patients/day	Not applicable PHCNP's home care caseload: 80–100 patients

^*^In the joint management model, a group of patients is managed jointly by the PHCNP and the physician partner. In the consultative management model, the PHCNP and the physician each manage a different group of patients and the PHCNP consults the physician as needed for patients in the group the PHCNP is following. The mixed model includes both joint and consultative patient management.

**Table 2:** Number of participants interviewed by profession and by case.

Participants	Case 1	Case 2	Case 3	Case 4	Case 5	Case 6	Total
PHCNP^*^	2	1	2	2	2	1	10
Physician partner	1	1	1	2	1	1	7
Management team member	2	1	2	2	2	2	11
Other interprofessional team member					1		1
Nurse and charge nurse	1		2	1	1		5

Total	6	3	7	7	7	4	34

^*^PHCNP: primary healthcare nurse practitioner; management team includes nurse and program managers and clinical nurse specialists.
